# Does corruption matter for FDI flows in the OECD? A gravity analysis

**DOI:** 10.1007/s10368-021-00496-4

**Published:** 2021-04-17

**Authors:** Tobias Zander

**Affiliations:** Schumpeter School of Business and Economics and European Institute of International Economic Relations (EIIW), University of Wuppertal, Wuppertal, Germany

**Keywords:** Foreign direct investment, Corruption, Gravity model, PPML, C33, D73, F21, F23

## Abstract

In this paper, the effect of corruption on foreign direct investment (FDI) flows is analyzed. The literature is thus far divided regarding the effects of corruption: One hypothesis argues that corruption greases the wheels of government and is therefore beneficial while the other hypothesis argues that it sands the wheels of government leading to suboptimal results in an economy. For the empirical analysis, a dataset consisting of bilateral FDI data from the OECD and the control of corruption measure from the World Governance Indicators of the World Bank is compiled. To further analyze the effects of corruption the Panama Papers revelation is used as a corruption increasing event and the implementation into law of the OECD Anti-Bribery Convention is used as a corruption decreasing event. Finally, the difference between corruption levels in the target and the origin country, will be examined. Then, a gravity model with dyadic and time-fixed effects is employed to analyze the data. Findings are ambiguous in that corruption is positively correlated with FDI inflows in the target country and negatively correlated with FDI inflows in the origin country. The Panama Papers variable shows strong evidence, that the release of the Panama Papers resulted in a drop in FDI flows. Therefore, it seems that corruption has complex country specific effects and that target and source countries have to adopt varying policies with regards to corruption. The general effect of corruption harms FDI flows, as shown by the Panama Papers revelation.

## Introduction

*“The more corrupt the state, the more numerous the laws.”*

Tacitus, The Annals of Imperial Rome (Tacitus and Grant 1959)

Foreign direct investment (FDI) has become increasingly relevant in past years. In 1995 FDI flows totaled $330 billion whereas in 2017 they had increased to $1.43 trillion (UNCTAD 2018). Many developing economies replace existing controls and restrictions over the entry of foreign multinational companies (MNCs) with new policies that are designed to attract and encourage FDI. Developing countries hope to benefit from FDI. Some of those benefits can be incoming capital, spillover effects associated with foreign technology as well as modern management skills and corporate governance (Alemu 2012).

But it is not just FDI that has gained in importance around the world. It is also corruption, or rather the fight against corruption, that has become more important over the last number of years. Recent corruption scandals show that corruption plays a big part in countries and economies around the world. For example, Volkswagens’ manipulation of the software in their diesel cars, the release of the Panama Papers, Brazil’s former presidents Dilma Rousseff and Luiz Inacio Lula da Silva corrupt dealings with the oil company Petrobras and South Korea’s President Park Geun-hye abuse of power to pressure conglomerates into millions of dollars of “donations” to just name a few (BBC News 2018a, b). Additionally, the abominable effects of corruption show especially in times of crisis. Regarding the Covid-19 pandemic, the United Nations Office on Crime and Drugs (UNODC) mentions for example the possibility of corrupt behaviour regarding the distribution of fiscal stimuli and rescue packages of governments around the world, especially who receives grants from these emergency funds (UNODC 2020a). Another issue raised with regard to the Covid-19 pandemic are the corruption risks related to vaccine development, production and distribution. Due to fast tracked research and development processes, opportunities for corruption arise due to conflicts of interest. Another area at risk for corruption is vaccine deployment as well as vaccine procurement; basically, the whole supply chain is at risk due to these extraordinary circumstances and sped-up processes and controls (UNODC 2020b).

Corruption was also a topic at the UN Security Council in 2018: Secretary-General António Guterres cited World Economic Forum estimates saying that the global cost of corruption is at least 5% of world GDP or $2.6 trillion (United Nations 2018). According to a World Bank estimate, businesses and individuals pay about 2% of global GDP or $1.5 trillion in bribes each year (World Bank 2017). Along these lines, Transparency International estimates that governments lose around $500 billion in tax revenues from businesses each year and further billions from individuals. These estimates should not be taken at face value as it is very hard to quantify the extent of damages caused by corrupt behavior. But it shows that corruption is treated as a very serious matter by major international organizations. Moreover, one cannot rule out that corruption in some countries facilitates a sometimes useful expansion of the shadow economy during critical periods – e.g. during a major recession – so that more people find a job and the overall effective real income could be raised, and poverty problems could possibly be alleviated in relatively poor countries. Such paradoxical real income effects are, however, not a key aspect considered in the subsequent analysis.

Compared to the early corruption research on FDI in the first decade of this millennium the data coverage and quality of data have improved for FDI as well as the estimation methods for the gravity model like the Poisson Pseudo Maximum Likelihood (PPML) estimator by Santos Silva and Tenreyro (2006). Building on these advantages this paper contributes to the literature by investigating corruption in a homogenous country group, namely the OECD, using high-quality bilateral FDI data from the OECD. By employing state of the art econometric modeling, i.e. a gravity model using PPML estimation and dyadic fixed effects as well as time fixed effects, new insights into the dynamics of corruption and FDI will be gained. Furthermore, the use of an event variable to model a corruption shock, i.e. the use of the revelation of the Panama Papers scandal as a shock that increases perceived corruption levels within this country group, brings new insights into the afore mentioned dynamics. Lastly, with the use of the OECD anti-bribery convention, a corruption curbing mechanism will be researched. As regards an analysis of the latter, some research has been done by Blundell-Wignall and Roulet (2017).

The reason for using only the OECD group of countries is due to the quality of data available but also to see what happens when one looks at a relatively homogenous group of primarily developed economies. Does corruption matter? Are subtle differences in corruption enough to affect FDI flows? Or are corruption levels low enough that companies do not need to care too much about it? Maybe there is just enough corruption for MNCs to take the risk and abuse these opportunities to their advantage? These are some of the questions that this paper is trying to answer.

Following the introduction is chapter 2 in which the theoretical framework as well as empirical findings regarding the nexus of FDI and corruption will be discussed. At the end of this chapter, hypotheses will be formed. Chapter 3 concerns the gravity model and its historical development from Newton’s law of gravity, to a model that explain trade flows and then to a model that explains FDI flows. Also, in chapter 3, there is a description of the data, control variables, model specification as wells as the estimation method used in the analysis while statistical challenges are also discussed. Chapter 4 presents the results of the estimation of the gravity model as well as a discussion of the empirical findings. Chapter 5 concludes with policy conclusion and an outlook on further research.

## Literature review / theoretical framework

### Corruption theory

The theoretical as well as the empirical literature on corruption shows a dichotomy when it comes to the effects of corruption. In the theoretical literature, there are two principle views on corruption, namely the ‘sand the wheels’ view and the ‘grease the wheels’ view. Sanding the wheels (of growth or FDI flows and so on) refers to the fact that corruption has a negative impact on the variable of interest (if the variable of interest is supposed to be “good” for an economy). Basically, corruption harms an economy and stops the economy from experiencing positive change over the years. Opposing this view is the grease the wheels view. Of course, the argument here is not that corruption suddenly is a positive for the economy. Rather, the idea is that corruption can be seen as a second-best case and can help under certain conditions to improve the status quo when compared with a case where corruption is not a possibility. Let us look at this view in more detail.

Méon and Sekkat (2005) summarize, that corruption could solve the issues arising from a malfunctioning administration. In particular, bribing corrupt officials might alleviate the problems of slowness of the public administration, rather poor skill levels on the part of civil servants, help to escape the consequences of some policies, and improve the quality of investment. Regarding slowness, Francis T. Lui (1985) showed in a queuing model that bribery could effectively speed up service, therefore, reducing the time spent in the queue. Bayley (1966) shows that corruption can improve the quality of civil servants in that it works as a kind of additional wage so that talented individuals are also attracted to possibly badly paying governmental jobs. Beck and Maher (1986) and Lien (1986) showed that, when comparing bribery to competitive bidding processes, there is no efficiency loss. In other words, the least cost firm will pay the highest bribe and therefore is awarded the price resulting in the generation of a desirable outcome (Beck and Maher 1986; Lien 1986). Leff (1964) argues that in the case of bad entrepreneurship policies, entrepreneurs effectively could implement their own favorable policies using corrupt measures such as bribes to incentivize civil servants to not implement the government’s policies. Leff (1964) continues that corruption may improve the quality of investment in that, for example, a bribe can be seen as a sort of insurance policy against the risk of expropriation or violence by the government.

Summarizing, corruption can, when faced with an inefficient and convoluted government and its policies and laws, lead to efficiency increases due to the possibility of circumventing the inefficacies produced by said government.

Switching now to the point of view of the sand the wheels hypothesis, Bardhan (1997) states:

In the second-best case made above, it is usually presumed that a given set of distortions are mitigated or circumvented by the effects of corruption; but quite often these distortions and corruption are caused or at least preserved or aggravated by the same common factors. The distortions are not exogenous to the system and are instead often part of the built-in corrupt practices of a patron-client political system

The grease the wheels hypothesis fails to recognize the enormous degree of discretion of many public officials regarding the regulatory burden (Kaufmann 1997). As Lambsdorff (2002) argues, corrupt public officials and politicians have a motivation of their own to create regulations. They do not need to be pushed to do so by private businessmen. Corruption gives public officials an incentive to create and impose regulations to maximize the bribes they get paid (Lambsdorff 2002). In the words of Kaufmann (1997):

This is one mechanism whereby corruption feeds on itself.

Boycko et al. (1995) stress that a bribe does not constitute a legal right that a court would protect nor does a bribe establish a contract that is enforceable in court.

Moreover, along with these arguments, Kaufmann and Wei (1999) investigate the effect of ‘speed-money’ and find evidence that suggests that, instead of saving time through bribes, entrepreneurs waste more time dealing with corrupt administrations. One may argue that from this perspective, transaction costs in markets are raised and this has a negative welfare effect in the respective country.

However, it is not only the argument of using bribes to speed up an inefficient government process which is addressed by the sand the wheels approach, the other arguments brought up above are addressed as well. When it comes to the quality of civil servants, Méon and Sekkat (2005) argue that officials also have an incentive to preserve their income from bribes by limiting the appointment of new and able officials to key positions. Regarding the efficiency argument in the bidding process, Kaufmann (1997) argues that corruption stands for a theft of public resources resulting in a decreased revenue stream for the treasury which can potentially impact macroeconomic stability as well as there being no guarantee that the winner is the most cost-efficient firm. Rose-Ackerman (1997) as well as Méon and Sekkat (2005) pick up this thought and argue that productive efficiency is not a requirement to win in a bidding process. Corruption favors those with no scruples and good connections (Rose-Ackerman 1997) and there is also the winner´s curse (Méon and Sekkat 2005). A related analytical approach with respect to markets points out that in the case of corruption with theft (meaning the public official does not turn over anything to the government and simply hides, for example the sale of a permit), competition between buyers helps spread corruption (Shleifer and Vishny 1993).

Moving on to escaping the consequences of some policies, here the grease the wheels view assumes that only “bad” policies are targeted and thereby overall efficiency could be improved. But “bad” policies for an entrepreneur or a company do not constitute inefficiencies or welfare loss for an economy. As Kaufmann (1997) mentions, some policies should not be escaped using bribes, for example, policies that prevent illegal logging of the rainforest or policies designed to protect the environment or air and water quality.

Regarding the argument that corruption may improve the quality of investment, it can be argued that corruption results in more public investment in unproductive sectors (Méon and Sekkat 2005). Corrupt officials favor projects that are one-of-a-kind, complex, and capital-intensive because corrupt payments are easier to conceal in these projects (Kaufmann 1997; Rose-Ackerman 1997). Therefore, defense projects or large infrastructure projects are preferred. Even more damaging are many unproductive projects that only enrich public officials and suppliers (Kaufmann 1997). Lastly, as corruption is illegal, the bribed officials have little incentive to truly commit to an agreement. Therefore, one can argue that bribes are not a safeguard against bad policies. On the contrary, corruption may as well lead to an increase in risks resulting from a weak rule of law (Méon and Sekkat 2005).

To sum up the theoretical views on corruption, there is an ongoing argument between seeing corruption as a second-best case that can, in some situations, lead to an efficiency gain on one hand and, on the other hand, seeing corruption as a condition that always results in a worse or unfavorable outcome. This ambiguity can also be seen in the findings of the empirical literature. Research has shown results that support both the sand the wheels as well as the grease the wheels view of corruption.

Some of the early empirical studies came from Wei (2000a, b). He finds evidence that corruption in a capital importing country distorts the composition of capital inflows towards foreign bank loans and away from FDI (Wei 2000a). Additionally, Wei (2000b) finds evidence that corruption reduces inward FDI stocks, acting comparably to an increase in taxation. Habib and Zurawicki (2002) find evidence that corruption as well as the difference in corruption between the host and source countries have a negative influence on FDI. Voyer and Beamish (2004) also find evidence in Japanese FDI supporting these earlier findings. Egger and Winner (2006) produce three results: 1) corruption, as measured via the Corruption Perception Index (CPI), has a negative impact on FDI, 2) corruption is an important factor for intra-OECD FDI but not for extra-OECD FDI, and 3) the impact of corruption for FDI, in general, has declined over the years. The authors argue that for horizontal intra-OECD FDI, trade impediments and factor cost differences are relatively low and that a change in perceived corruption could result in MNCs deciding to engage in trade rather than horizontal FDI.

Al-Sadig (2009) finds evidence that the corruption level has negative effects on FDI inflows but this effect loses significance once institutional quality is introduced in the regression. The author concludes that sound institutions are more important for attracting FDI than corruption levels. Alemu’s (2012) findings also support earlier studies. Belgibayeva and Plekhanov (2015): hypothesize that FDI is not homogenous and depends on the level of corruption in the host country. They use Eurostat data from 1992 to 2011 for EU countries, Turkey and FYR Macedonia. Their evidence suggests that, overall, corruption deters foreign direct investment. They also find that the level of corruption affects the composition of FDI meaning that reducing corruption then attracts more FDI from less corrupt countries.

Most of these earlier studies found support for the sand the wheels hypothesis. In contrast, more recent studies often find evidence that corruption is indeed a facilitator for FDI. Bellos and Subasat (2011), for example, and the follow-up study of Subasat and Bellos (2013) employ a gravity model to investigate the connection between FDI and corruption. Their results point towards the grease the wheels hypothesis, meaning that a decrease in corruption levels would lead to a decrease in FDI inflows. Barassi and Zhou (2012) employ both parametric and non-parametric analyses and find that, after controlling for the location selection process of MNCs, corruption has a positive impact on FDI stocks. They also find that, in their non-parametric analysis, the effect of corruption is heterogeneous and depends on the level of FDI stock. Finally, Blundell-Wignall and Roulet (2017), using dyadic fixed ordinary least squares (OLS) estimation and GMM estimators, find that corruption, in general, has either an insignificant or a positive effect on FDI.

There are also studies from behavioral economics focusing on exploring the question on why there even is corrupt behavior. As Lambsdorff (2012) puts it: “Homo economicus is either horribly corrupt, because he feels no moral impediments, regards all temptations to be legitimate and takes advantage of risks of punishment being commonly low. Or Homo economicus is averse to corruption, because corruption is arduous to enforce. Homo reciprocans provides a better app-roach to understanding corruption. As now widely evidenced in experimental research, humans are sometimes willing to reciprocate a bribe but they also devote resources to an altruistic punishment of bribe-takers and like to serve their principals.” Behavioral economics also helps to gain an understanding regarding the efficacy of anti-corruption measures. Lambsdorff explores this in another paper where he studies six anti-corruption measures and illustrates why these measures, even though they are seen as best practice, show mediocre results at best and are often counterproductive and inefficient (Lamsbdorff 2015). Although this strand of literature is very interesting and delivers many answers this study will not further pick up on this.

Although all these studies vary in their scope, country selection, model, and estimation method, one can say that, overall, there is more empirical support for the sand the wheels hypothesis. Considering theory as well as empirical findings over the years, one can argue that corruption could have an ambiguous effect dependent on the prevalent characteristics of the countries included in the dataset. Nevertheless, we will adopt the view that higher levels of corruption are detrimental for attracting FDI.

### FDI theory and main FDI determinants

Regarding why companies engage in FDI, there have been several theories over the years (for an extensive review, see e.g. Faeth (2009)). One of the earliest theories was the approach of Dunning. He first introduced the concept of the eclectic paradigm of international production in 1976. Dunning wanted to create a holistic framework that is able to identify and evaluate the factors that influence the initial decision and act of foreign production and the growth of such production. He chose to label his theory eclectic as several strands of economic theory are needed to explain the transnational activities of enterprises (Dunning 1988).

In short, the eclectic paradigm states that a combination of the following three advantages is necessary for an MNC to enter into a foreign market: Ownership-specific advantages (O-advantages), location-specific advantages (L-advantages), and internalization-specific advantages (I-advantages) (Hermannsdottir 2008; Dunning 1988). FDI gravity analysis often focuses on the location-specific advantages of the target and host country.

As Faeth (2009) puts it: “Empirical studies testing the OLI framework have found FDI to be determined by a combination of ownership advantages, market size and characteristics, factor costs transport costs, protection and other factors including regime type, infrastructure, property rights and industrial disputes.”

In an early study of the empirical literature regarding FDI determinants, Blonigen (2005) identifies exchange rate effects, taxes, institutions, trade protection and trade effects as main determinants of FDI. He also points out the difficulty in developing a general equilibrium model for FDI, since FDI patterns appear to be more complex than trade patterns, since there seems to be two general motivations for FDI: horizontal FDI, which aims to access markets in the face of trade frictions and vertical FDI which is to access low wages for part of the production process. Building on these, in papers by Carr et al. (2001) as well as Bergstrand and Egger (2007), theoretical models have been developed that suggest additional possible factors for determining FDI patterns. As Blonigen and Piger (2014) point out, standard gravity variables capture horizontal FDI patterns, but for explaining vertical FDI patterns these additional control variables are needed (see e.g. Baltagi et al. 2007).

Both Blonigen and Piger (2014) as well as Eicher et al. (2012) study robust determinants of FDI. Key gravity variables according to Blonigen and Piger (2014) are real GDP, distance, a common language and colonial relationships, trade openness of the host country, customs union, regional trade agreements as well as endowment differences across host and source countries. Eicher et al. (2012) find that a lack of corruption, ethnic tension, as well as the corporate tax rate are additional robust determinants of FDI flows. The authors also study robust determinants for only OECD countries. Here they find that for OECD country-pairs a common language, membership of EFTA, and military influence in governance lose relevance as determinants, whereas higher levels of development, government instability, financial risk, and bureaucratic efficiency gain in relevance. Key gravity equation parameters are not affected and remain robust determinants (Eicher et al. 2012).

### Hypotheses

As previously stated, we will follow the sand the wheels arguments in that corruption is seen to be detrimental to FDI. Therefore, the main hypothesis of this paper is as follows:

1. Corruption has a significant and negative effect on FDI flows.

We will employ multiple methods to try to capture these corruption effects. Therefore, the main hypothesis has to be specified and adjusted accordingly:

A. The higher the corruption levels are, the smaller the FDI flows. Corruption levels will be measured by the Control of Corruption (COC) Index of the World Bank´s Worldwide Governance Indicators. This index is used over the corruption index of Transparency International (TI) because the TI index is only comparable over time from 2011 onwards (https://www.transparency.org/files/content/pressrelease/2012_CPIUpdatedMethodology_EMBARGO_EN.pdf, last accessed 07.09.2020).
B. The higher the difference between corruption levels (see e.g. Qian and Sandoval-Hernandez (2016) or Habib and Zurawicki (2002)) of the target and origin country, the smaller the overall FDI flows for this country-pair.

As regards B., one may argue that a similar level of corruption in the host country and the home country represents a similarity in the respective economic systems which, in turn, reduces investors’ information costs abroad and therefore a similar level of corruption should stimulate FDI flows.

According to Kaufmann et al. (2010), the COC index is: “capturing perceptions of the extent to which public power is exercised for private gain, including both petty and grand forms of corruption, as well as "capture" of the state by elites and private interests.” The index ranges from −2.5 to 2.5 with 2.5 meaning no perceived corruption at all and −2.5 being the highest amount of perceived corruption. As this is unintuitive, the index will be rescaled to that it ranges from 0 to 5 with 0 being the lowest corruption levels and 5 being the highest. One can also note that the COC Index does not have values for the years 1997, 1999, and 2001. We approximate these values by using the average of the years before and after.

The revelation of the Panama Papers scandal as an event of increasing overall corruption and also an increased overall perception of corruption will be used to study the effects of such an event on FDI. This leads to the following hypothesis:

C. The reveal of the Panama Papers is expected to have a negative effect thereby decreasing overall FDI flows.

Finally, the implementation into law of the OECD Anti-Bribery Convention as a way of researching the effect of an anti-corruption measures will be analyzed: According to the OECD, “The OECD Anti-Bribery Convention establishes legally binding standards to criminalize bribery of foreign public officials in international business transactions and provides for a host of related measures that make this effective” (OECD Website, http://www.oecd.org/corruption/oecdantibriberyconvention.htm, last accessed 07.09.^2020^).

Thus, the OECD Anti-Bribery Convention – if implemented—is regarded as a corruption reducing action as it makes bribery, a significant element of corrupt behavior, punishable by law thereby increasing the ramifications for those caught engaging in such corrupt behavior (see Blundell-Wignall and Roulet (2017) for extensive research regarding the effects of the OECD Anti Bribery Convention):

D. The implementation into law of the OECD Anti-Bribery Convention in the target country, as a corruption reducing event, will have a positive effect on FDI inflows.

## Methodology

### The gravity model

The first time the concept of a gravity model appeared in economics was in 1889 when Ravenstein used it to model migration patterns in the UK (Anderson 2011). Then, in 1962 Tinbergen used it the first time to model trade flows and in its most basic form it can be written as follows:

1 $$\log X_{ij} = c + b_{1} \log GDP_{i} + b_{2} \log GDP_{j} + b_{3} \log \left( {distance_{ij} } \right){\text{ + e}}_{i}$$

where X_ij_ indicates imports from country i to country j, GDP represents each country’s respective GDP, the distance between them, distance_ij_, is an observable proxy for trade costs, e_ij_ is the error term, c is a regression constant, and b_1_ to b_3_ are coefficients to be estimated. From here the reason why it is called a gravity model becomes clearer as Eq. (1) resembles Newton’s law of gravity^1^ which states that every object attracts every other object in the universe with a force which is directly proportional to the product of their masses and inversely proportional to the square of the distance between their centers. In economic terms the force becomes exports, the mass becomes GDP and the squared distance becomes distance. In other words, bigger countries trade more and countries that are further apart from each other trade less (Shepherd 2016).

The next step in the evolution of the gravity model occured when Anderson and van Wincoop published their famous ‘gravity with gravitas’ paper in 2003. Essentially, this model is a demand function where consumers have ‘love of variety’ preferences meaning that their utility increases both from consuming a wider range of varieties or from consuming more of a given product variety (Shepherd 2016). Anderson and van Wincoop’s theoretical results show that bilateral trade is determined by so-called multilateral trade-resistance terms, in other words, bilateral trade is determined by relative trade costs. This means that exports from country j to country i depend on all export markets and that imports from country i to country j depend on trade costs across all possible suppliers. To give an example, Belgium and the Netherlands, which are bordered by two large trading economies, namely France and Germany, and by each other, will trade less between themselves than if they were surrounded by vast mountains or by oceans. This leads to the theoretically-funded gravity equation:

2 $$X_{ij} = \frac{{Y_{i} Y_{j} }}{Y}*\left( {\frac{{t_{ij} }}{{\prod\nolimits_{{_{i} }} {{\text{P}}_{j} } }}} \right)^{{1 -\upsigma }}$$

where Y represents world GDP, Y_i_ and Y_j_ the GDP of country i and j respectively, t_ij_ represents the cost in j of importing the good from i, σ > 1 denotes the elasticity of substitution and Π_i_ and Ρ_j_ denote country i’s outward and country j’s inward multilateral resistance terms (Bacchetta et al. 2012).

In log-linearized form, one thus gets the following Eq. (3):

3 $$\ln X_{ij} = c + b_{1} \ln Y_{i} + b_{2} \ln Y_{j} + \left( {1 - {\upsigma }} \right)\ln {\uptau }_{ij} + b_{3} \ln \Pi_{i} + b_{4} \ln {\text{P}}_{j} + e_{ij}$$

The difficulty with Eq. (3) is that the multilateral resistance terms are not directly observable. To solve this problem a commonly used option is the use of country fixed-effects for importers and exporters (Bacchetta et al. 2012).

The next step in the evolution of the gravity model came from Larch et al. (2017). In their paper, the authors laid the theoretical foundation for the use of the gravity model not just for trade analysis but also for FDI analysis. The authors get the following FDI gravity system for the steady-state with (4) being the function for the FDI stock and (5) and (6) representing multilateral resistance terms. The detailed derivation of this system can be found in Larch et al. (2017).

4 $${FDI}_{ij}^{stock,value}=\frac{\upbeta {\upphi }^{2}{\upeta }_{i}^{2}{\updelta }_{M}}{1-\upbeta +\upbeta {\updelta }_{M}}{\upomega }_{ij}^{\upxi }\frac{{E}_{i}}{{P}_{i}}\frac{{Y}_{j}}{{M}_{i}}$$

5 $${\mathrm{P}}_{i}={\left[\sum_{j=1}^{N}{\left(\frac{{t}_{ji}}{{\Pi }_{j}}\right)}^{1-\sigma }\frac{{Y}_{j}}{Y}\right]}^{\frac{1}{1-\sigma }}$$

6 $${\Pi }_{j}={\left[\sum_{i=1}^{N}{\left(\frac{{t}_{ji}}{{P}_{j}}\right)}^{1-\sigma }\frac{{E}_{i}}{Y}\right]}^{\frac{1}{1-\sigma }}$$

(4), (5) and (6) from Larch et al. (2017).

E_i_: the size of the country of origin
Y_j_: the size of host country
W_ijt_: FDI barriers
P_i_: inward multilateral resistance terms
Π_j_: outward multilateral resistance terms

Equations (5) and (6) represent the inward and outward multilateral resistance terms for FDI, respectively (the phrase multilateral resistance terms come from Anderson and van Wincoop in their 2003 paper; also used in Eq. (2) but with a different definition). Our main interest lies with Eq. (4) as it reveals several interesting relationships. Firstly, it shows that FDI is directly linked to the size of the country of origin, as measured by expenditure E_i_. Secondly, it shows the positive connection between FDI and the size of the host country, measured by nominal output Y_j_ (this fits with the intuitive gravity model whereby the “mass” of the countries is a significant influence on the “attractive force”). Thirdly, ω_ij_ captures FDI barriers and thereby reveals the negative relationship between FDI and said FDI barriers. Additionally, Eq. (4) shows the link between FDI and trade via the multilateral resistance terms (MRTs). In detail, higher inward MRTs of the origin country should lead to less FDI abroad in general and at the destination country in particular. Interestingly, there is no outward multilateral resistance term in Eq. (4). Larch et al. (2017) justify this with the fact that technology capital is non-rival, i.e. in contrast to goods that are sold from i to j and then cannot be used elsewhere, the technology of country i that is used in country j can also be used elsewhere. And lastly, this equation also shows that the value of the FDI stock of country i in country j depends negatively on the amount of technology capital in country i (Larch et al. 2017).

Conveniently, this FDI gravity system can be estimated empirically using the standard fixed effects techniques of the trade gravity literature (Larch et al. 2017). To transform Eq. (4) into an econometric equation, the authors propose to first model the FDI frictions ω_ij,t_. To this end, they suggest decomposing the frictions into four categories:

1. Characteristics of the source country, such as corporate tax rate, corruption, red tape, etc.
2. Characteristics of the host country, such as corruption, corporate tax rate, internal tensions, etc.
3. Time-invariant bilateral characteristics for the two partners, such as distance, common official language, colonial relationships, etc.
4. Time-varying bilateral determinants of FDI, such as regional trade agreements, customs union, etc.

These determinants are based on the studies of Blonigen and Piger (2014) and Eicher et al. (2012), which have been discussed in a previous chapter. It is worth noting that for a dataset with only OECD countries, different determinants are relevant, e.g. EFTA membership loses its relevance when looking at only OECD countries. This is due to very little variation in these variables across OECD countries (Eicher et al. 2012). Therefore, we only use the Eurozone as a time-varying bilateral determinant of FDI (point 4).

In sum, the gravity model of trade has been proven to be a useful tool in analyzing international trade. Larch et al. (2017) also showed that it can be used to analyze FDI flows. Recent studies (see, e.g., Bruno et al. (2016), Blundell-Wignall and Roulet (2017); and Baier (2020)) have successfully used the gravity model to explain foreign investment flows and for this and the reasons above, it is used in this paper as well.

### Data and control variables

In this study, bilateral FDI flow data from the OECD is used for the years 1996 to 2017. The bilateral data is available from 1985 but, due to data limitation, mainly resulting from the COC Index only starting in 1996, the timespan for the dataset is shortened.

**Fig. 1:**
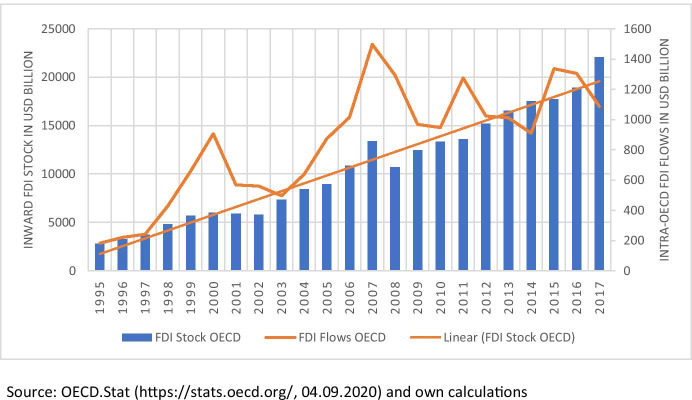
FDI developments in the OECD, from 1995 to 2017

Figure 1 shows the development of the total OECD inward FDI stock in billions of US Dollars and the total yearly FDI flows in billions of US Dollars. In 1995, the total FDI Stock is roughly at about US$4,000 billion whereas in 2018 it is almost at US$22,000 billion. That is more than a five-fold increase over 23 years. Total intra-OECD FDI flows follow a similar trend but are of course more volatile than the stock. In 1995, there were roughly US$200 billion in FDI flows increasing to roughly US$1,100 billion in 2017. Needless to say, that FDI does indeed play a very big role in OECD economies. The figure also shows a very clear, linear, upward trend for both stock and flows. This is in line with the trend mentioned in the introduction of FDI flows increasing worldwide from 330 billion in 1995 to 1.43 trillion US Dollars in 2017 (UNCTAD 2018).

The OECD has a total of 36 member countries. This when combined with the timespan of 22 years results in a total possible number of 27,720 observations. Due to missing values, this number drops to 15,408. Table 1 shows the summary statistics concerning the dependent as well as the independent variables.

**Table 1:** Summary statistics with mean, standard deviation (sd), minimum and maximum value

	Count	Mean	sd	Min	Max
inflow	15,408	1152.338	5341.779	0	172,740
ln_target_gdp	15,408	12.89243	1.585268	8.935393	16.79051
ln_origin_gdpp	15,408	12.92831	1.583329	8.645722	16.79051
ln_pop_target	15,408	2.726963	1.54765	−1.305157	5.784278
ln_pop_origin	15,408	2.673425	1.527832	−1.305157	5.784278
ln_agglo_l1	15,408	11.67986	1.527454	5.295098	15.70052
ln_patents_target	15,408	7.546825	2.248416	2.70805	12.85892
ln_patents_origin	15,408	7.607631	2.176204	2.564949	12.85892
target_tax	15,408	27.81796	7.492964	9	56.8
origin_tax	15,408	28.10505	7.260312	9	56.8
openness	15,408	0.8679761	0.4753944	0.1865374	3.166917
ea_dummy	15,408	0.1516745	0.3587167	0	1
coc_l1_target	15,408	1.221463	0.8031797	0.030009	3.021816
coc_l1_origin	15,408	1.126718	0.7776764	0.030009	3.265936
panama_event	15,408	0.1102025	0.3131521	0	1
oecd_ab_target	15,408	0.9000519	0.2999405	0	1
coc_diff_l1	15,408	0.9062835	0.6617513	0.0003068	3.041567

Source: Own calculations

The control variables used are based on the previous studies by Faeth (2009), Blonigen and Piger (2014) as well as Eicher et al. (2012). The reason for not using GDP per capita but instead using the total population is because GDP per capita and the corruption variables show a very high correlation.^2^ Therefore, to avoid potential multicollinearity issues, GDP per capita is not used in this analysis. In the Appendix, there are two models, based on model (4) and (8), that use GDP per capita as a form of robustness check. The effect on the estimated value of the COC variable is, however, fairly small. Table 2 shows the correlation of the dependent variable inflow and the independent variables.

**Table 2:** Correlation table of the dependent and the independent variables

	inflow
inflow	1
ln_target_gdp	0.174***
ln_origin_gdp	0.155***
ln_pop_target	0.114***
ln_pop_origin	0.0956***
ln_agglo_l1	0.226***
ln_patents_target	0.132***
ln_patents_origin	0.126***
target_tax	0.115***
origin_tax	0.0941***
openness	0.0218**
ea_dummy	0.0445***
coc_l1_target	−0.0830***
coc_l1_origin	−0.0990***
panama_event	0.0163*
oecd_ab_target	0.0371***
coc_diff_l1	−0.120***

Source: Own calculations, full table in Appendix

* *p* < 0.05, ** *p* < 0.01, *** *p* < 0.001"

Every independent variable is significantly correlated with the dependent variable without having a high correlation, introducing potential issues of multicollinearity.

Table 3 provides an overview of the dependent and independent variables that will be used.

**Table 3:** Description of *dependent* and independent variables

Variable	Definition	Source
*Inflow*	*Bilateral FDI intra-OECD flows*	*OECD FDI database; BMD3 data 1985–2012, BMD4 data 2013–2017*
ln_target_gdp	GDP of FDI Target Country, in current USD	World Bank
ln_origin_gdp	GDP of FDI Origin Country, in current USD	World Bank
ln_pop_target	Total Population of the Target Country, in millions	OECD
ln_pop_origin	Total Population of the Origin Country, in millions	OECD
openness	Total import plus total export of FDI target country, divided by its GDP	World Bank
target_tax	General FDI target country corporate tax rates, including average/typical local taxes	Mintz and Weichenrieder (2010); KPMG (2020)
origin_tax	General FDI origin country corporate tax rates, including average/typical local taxes	Mintz and Weichenrieder (2010); KPMG (2020)
ln_patents_target	Patent applications by residents of the target country	World Bank
ln_patents_origin	Patent applications by residents of the origin country	World Bank
ln_agglo_l1	Agglomeration effects (inward FDI stock) in the target country lagged by 1 year	OECD
ea_dummy	Dummy variable that takes the value 1 if both countries are part of the eurozone	European Commission
coc_l1_target	The level of corruption in the target country, lagged by 1 year. Rescaled to 0 – 5 (5 being maximum corruption)	World Bank WGI Project by Kaufmann, Kray and Mastruzzi
coc_l1_origin	The level of corruption in the origin country, lagged by 1 year. Rescaled to 0 – 5 (5 being maximum corruption)	World Bank WGI Project by Kaufmann, Kray and Mastruzzi
Coc_diff_l1	Difference between host and source country level of corruption	World Bank WGI Project by Kaufmann, Kray and Mastruzzi
panama_event	Event dummy that takes the value 1 when the Panama Papers were revealed (2016)	
oecd_ab_target	Dummy variable for the target country, that takes the value 1, once the OECD Anti Bribery Convention was implemented into law	OECD

The effects that are to be expected for these control variables based on the theoretical and empirical literature previously discussed, are as follows:

1. Market size, measured by GDP and the population, has a positive and significant effect on FDI flows.
2. Corporate taxes have a negative impact on FDI flows.
3. Trade openness, representing a measure for the ease of trade, will have a positive effect on FDI flows. In other words, a country open for trade will attract more FDI flows.
4. Agglomeration effects are significant and positive contributors to FDI flows, meaning that countries with an already high FDI stock will attract more FDI flows than countries with a smaller FDI stock.
5. The innovative capacity of an economy is expected to have a positive effect on FDI flows. The number of patent applications in a country is used to measure this.
6. Eurozone dummy: it is expected that when both countries are in the Eurozone they have more FDI flows (see e.g. Zhang (2004) for an early study on the effects of the European Monetary System on intra-EU FDI; for another related study, but with an EU dummy instead of Eurozone dummy, see Bruno et al. (2016))

Combining theory, the variables of interest and control variables then results in the following specification of the gravity model:

7 $$FDIflows_{odt} = c + b_{1} \ln X_{ot} + b_{2} X_{dt} + b_{3} X_{o(t - 1)} + b_{4} X_{d(t - 1)} + \delta_{od} + \tau_{t} + \varepsilon_{odt}$$

c: regression constant
X_ot_: origin country time variant characteristics
X_o(t−1)_: lagged origin country time variant characteristics
X_dt_: destination/target country time variant characteristics
X_d(t−1)_: lagged destination/target country time variant characteristics
δ_od_: time invariant dyadic fixed effects
τ_t_: time fixed effects
ε_odt_: error term

Lastly, several statistical challenges have to be addressed.

- Zero values in the data: 5,865 of 15,408 observations (thus, circa 38% of observations are zeroes). Some of the 15,408 observations are dropped in the later analysis to ensure the existence of estimates.
- Heteroskedasticity: Breusch Pagan / Cook Weisberg testing reveals the presence of heteroskedasticity.
- Endogeneity: to avoid potential endogeneity problems regarding the dependent variable the corruption variables, corruption variables are lagged by 1 year.
- Missing values are dealt with by listwise deletion.
- Negative values are set to zero.
- Stationarity is not an issue here as our N (15,408) is much larger than our T (21).

The solution to the statistical challenges presented here is the Poisson Pseudo Maximum Likelihood estimator (PPML) by Santos Silva and Tenreyro (2006).

### Estimation method

There are many ways to estimate the gravity model. For a detailed discussion see Kareem et al. (2016). For the analysis, we will use the Poisson Pseudo-Maximum Likelihood (PPML) estimator by Santos Silva and Tenreyro (2006). The PPML estimator is an analysis often-used estimator in modern trade and FDI gravity analysis due to its superior performance over the OLS estimator. Shepherd (2016) points out that in the case of a multiplicative error term in the theoretical gravity model, log-linearization in the presence of a heteroscedastic error term violates the first OLS assumptions and leads to inconsistent estimates. Santos Silva and Tenreyro (2006) provide a solution to this problem. They show that that the PPML estimator is robust to different patterns of heteroskedasticity and produces consistent estimates of the non-linear model. Basically, PPML estimates the gravity equation in levels instead of taking logarithms (Kareem et al. 2016).

For FDI analysis, the OLS estimator has been the most used estimator (Larch et al. 2017). When using OLS, standard procedure would be either to delete zero trade flows or one would simply give zero values the place holder value of $1 (in comparison to the usual millions of dollars of FDI flows, an insignificant value) as the OLS model is estimated using the logarithm of the FDI flows and the logarithm of 0 is not defined. Of course, deletion as well as assigning a nominal $1 value introduces some bias into the dataset (Welfens and Baier 2018). However, more recently PPML has seen increased usage. Biro et al (2019) decided to test the performance of PPML vs OLS with regards to FDI. They find that the PPML estimator gives a better fit to the data, yielding unbiased, consistent, and efficient results when compared to the OLS estimator.

Shepherd (2016) points out several advantages of the PPML estimator. Firstly, the PPML estimator includes observations for which the observed value is zero. Secondly, it is consistent in the presence of fixed effects. And thirdly, like Kareem et al. (2016) mention, the PPML estimator takes account of observed heterogeneity.

As the analysis uses fixed effects, the dataset has a large number of zeroes and we observe heteroskedasticity (Breusch pagan / Cook Weisberg test), the logical conclusion is to use the PPML estimator as it is best equipped to deal with these issues and is proven to be consistent and performs better when compared to OLS. These findings in combination with the arguments by Shepherd (2016) and Kareem et al. (2016) as well the use of PPML in recent studies (e.g. Biro et al. (2019) and Baier (2020)) is enough evidence for us to use the PPML estimator as our main estimation method (OLS estimation will be used for robustness checks).

## Results

As mentioned in previous chapters, the models will be estimated using the PPML estimator as well as country pair and time fixed effects. Two different ways of using the COC Index were implemented for this analysis, the first one is to simply use the value of the index for the target and source country lagged by 1 year to avoid possible endogeneity. The second way is to use the difference in corruption levels of the target and source country, also lagged by 1 year for the same reasons. In models (1) to (4), the COC Index will be used, in models (5) to (8) the difference in corruption index will be used. The event variable for the Panama Papers scandal, panama_event, will be introduced in models (2) and (4) for the COC analysis, and models (6) and (8) for the corruption difference analysis. The OECD Anti-Bribery Convention dummy, OECD_ab, will be introduced in models (3) and (4), as well as (7) and (8). Model (1) is the baseline model with the usual gravity variables and the COC measure. Model (2) then introduces the Panama Papers event dummy. In model (3) the Panama event dummy is switched for the OECD Anti-Bribery event dummy. Then in model (4), both dummy variables are added. Models (5) to (8) follow the same logical structure only that here, the corruption measure is the corruption distance.

Table 4 shows the result of the PPML estimation with dyadic and time fixed effects. Models (1) – (4) employ the COC index as the corruption measure. The standard gravity variables are significant and have the expected sign, thereby validating our model. Specifically, the GDP of the target country (1% significance level) and the population (5% significance level in msodel (1) and (2), 10% significance level in models (3) and (4)), as well as agglomeration effects (10% significance in models (1) and (2)) and trade openness^3^ (1% significance level), show statistical significance and the expected positive sign. Surprisingly, the results for the tax rates of the target country as well as the country of origin show no significance. The patent variable is also not significantly different from zero in any of the models. The dummy for the Eurozone, on the other hand, shows significance at the 5% level in all four estimated models. It also has the expected positive sign, indicating that when both countries are part of the Eurozone, FDI flows between them increases.^4^ Moving on to our variables of interest, the corruption variables, both COC variables for the target and origin country are significant at the 5% level in all four models, the Panama event dummy is also significant (10% level) in the models it was estimated (i.e. models (2) and (4)). The OECD Anti-Bribery dummy is not significant. The COC variables have interesting signs, deviating from our expectations. It seems that for target countries of FDI, the COC is positively correlated (semi-elasticities between 0.67% and 0.63%), whereas for host countries it is negatively correlated (semi-elasticities of −0.42%). The Panama event dummy has the expected negative sign and a semi-elasticity of −36% on FDI flows between country pairs.

**Table 4:** PPML estimation with COC variable

Variables	(1)	(2)	(3)	(4)
	Model 1	Model 2	Model 3	Model 4
**ln_target_gdp**	1.082***	1.082***	1.032***	1.032***
	(0.352)	(0.352)	(0.354)	(0.354)
ln_origin_gdp	0.322	0.322	0.318	0.318
	(0.342)	(0.342)	(0.344)	(0.344)
**ln_pop_target**	3.921**	3.921**	4.119**	4.119**
	(1.883)	(1.883)	(1.918)	(1.918)
ln_pop_origin	1.889	1.889	1.883	1.883
	(1.753)	(1.753)	(1.751)	(1.751)
**ln_agglo_l1**	0.233*	0.233*	0.208	0.208
	(0.128)	(0.128)	(0.131)	(0.131)
ln_patents_target	0.216	0.216	0.215	0.215
	(0.139)	(0.139)	(0.141)	(0.141)
ln_patents_origin	0.0231	0.0231	0.0215	0.0215
	(0.124)	(0.124)	(0.124)	(0.124)
target_tax	−0.00558	−0.00558	−0.00404	−0.00404
	(0.0100)	(0.0100)	(0.00980)	(0.00980)
origin_tax	0.000306	0.000306	0.000261	0.000261
	(0.0105)	(0.0105)	(0.0104)	(0.0104)
**openness**	2.035***	2.035***	1.970***	1.970***
	(0.375)	(0.375)	(0.369)	(0.369)
**ea_dummy**	0.273**	0.273**	0.234**	0.234**
	(0.120)	(0.120)	(0.119)	(0.119)
**coc_l1_target**	0.511**	0.511**	0.489**	0.489**
	*(0.201)*	*(0.201)*	*(0.204)*	*(0.204)*
**coc_l1_origin**	−0.542**	−0.542**	−0.544**	−0.544**
	*(0.258)*	*(0.258)*	*(0.258)*	*(0.258)*
**panama_event**		−0.439*		−0.435*
		*(0.226)*		*(0.226)*
*oecd_ab_target*			*0.305*	*0.305*
			*(0.195)*	*(0.195)*
Observations	14,626	14,626	14,626	14,626
R-squared	0.562	0.562	0.562	0.562

Robust standard errors in parentheses

All models are estimated using dyadic and time fixed effects. They have been omitted for brevity

Corruption variables in italic, statistically significant variables in bold

*** *p* < 0.01, ** *p* < 0.05, * *p* < 0.1

Models (5) to (8) follow the same principle as models (1) to (4) except for the corruption variable and results are shown in Table 5. Here, we now use the corruption difference of the host and the target country as the corruption measure. The use of this variable produces additional findings. Our gravity variables for the target country, GDP, population (significance level of 10% for models (5) and (6), 5% for models (7) and (8)), openness, agglomeration effects and the eurozone dummy (5% significance level in models (5) and (6), 10% significance level in models (7) and (8)) remain roughly the same^5^ (openness coefficient slightly less). The same when it comes to the coefficients of these variables. The newly introduced corruption distance variable shows no significance in any of the models, the Panama event dummy remains statistically significant at the 10% level and negative (roughly the same coefficient as well). In models (7) and (8), we introduce the OECD Anti-Bribery dummy and here it is significant at the 10% level. The coefficient is positive (semi elasticity of 39%) indicating a positive effect on FDI inflows for the target country, once the OECD Anti Bribery Convention was implemented into law. One will not necessarily expect the impact of a lagged variable here since political debate will have an early signaling effect on investors – prior to legal changes being fully implemented.

**Table 5:** PPML estimation with corruption difference variable

Variables	(5)	(6)	(7)	(8)
	Model 5	Model 6	Model 7	Model 8
**ln_target_gdp**	0.897***	0.897***	0.852***	0.852***
	(0.328)	(0.328)	(0.329)	(0.329)
ln_origin_gdp	0.421	0.421	0.416	0.416
	(0.372)	(0.372)	(0.374)	(0.374)
**ln_pop_target**	3.555*	3.555*	3.777**	3.777**
	(1.879)	(1.879)	(1.911)	(1.911)
ln_pop_origin	1.405	1.405	1.397	1.397
	(1.779)	(1.779)	(1.776)	(1.776)
**ln_agglo_l1**	0.224*	0.224*	0.197	0.197
	(0.130)	(0.130)	(0.134)	(0.134)
ln_patents_target	0.157	0.157	0.158	0.158
	(0.141)	(0.141)	(0.142)	(0.142)
ln_patents_origin	0.0911	0.0911	0.0903	0.0903
	(0.125)	(0.125)	(0.125)	(0.125)
target_tax	−0.000140	−0.000140	0.00138	0.00138
	(0.0108)	(0.0108)	(0.0105)	(0.0105)
origin_tax	−0.00209	−0.00209	−0.00211	−0.00211
	(0.0110)	(0.0110)	(0.0108)	(0.0108)
**openness**	1.847***	1.847***	1.786***	1.786***
	(0.370)	(0.370)	(0.363)	(0.363)
**ea_dummy**	0.259**	0.259**	0.217*	0.217*
	(0.128)	(0.128)	(0.127)	(0.127)
*coc_diff_l1*	*0.233*	*0.233*	*0.227*	*0.227*
	*(0.148)*	*(0.148)*	*(0.148)*	*(0.148)*
**panama_event**		−0.409*		−0.406*
		*(0.227)*		*(0.227)*
**oecd_ab_target**			0.329*	0.329*
			*(0.193)*	*(0.193)*
Observations	14,626	14,626	14,626	14,626
R-squared	0.560	0.560	0.561	0.561

Robust standard errors in parentheses

All models are estimated using dyadic and time fixed effects. They have been omitted for brevity

Corruption variables in italic and statistically significant variables in bold

*** *p* < 0.01, ** *p* < 0.05, * *p* < 0.1

### Empirical findings

As regards the first hypothesis A, that higher corruption levels result in lower FDI flows, has two results and key results, respectively. The analysis shows that the effect of corruption here is different for source and target countries of FDI. For target countries, a positive correlation was found in models (1) to (4) (semi-elasticities between 0.63% and 0.67% dependent on model specification) whereas for the source country a negative correlation (semi-elasticities of −0.42%) was found. The results are therefore ambiguous and hypothesis A cannot be corroborated – the finding that corruption in target countries shows a positive correlation could be interpreted as follows: corruption-inclination in target countries effectively allows to reduce foreign investors’ risk premium because corruption can be used to influence the bureaucracy and investor-related regulations in a favorable way; note that one cannot rule out that a more FDI friendly “effective business climate” also stimulates more investment and higher R&D expenditure ratios by domestic firms in the respective home countries which, in turn, will stimulate more FDI inflows from abroad. Hence a direct and indirect FDI link could be relevant – only further research could shed more light on these two channels.

The finding that source countries’ corruption levels have a negative effect on FDI could potentially point to the problem that corruption is associated with the risk of an ad-hoc intervention of government and bureaucratic agencies vis-à-vis all or most multinational companies which therefore will aim to reduce overall investor risk – and this could include reduced R&D expenditures on the part of firms: with a lower R&D expenditure ratio the ability of firms to generate a critical minimum level of owner-specific technological advantage could be restricted and the consequence is a reduction of FDI.

For hypothesis B there is no evidence since we do not get statistically significant results thereby are not able to state with confidence that the point estimate for the corruption distance is different from zero.

The results for hypothesis C on the other hand are clear. In all models where the panama event dummy was used (Models (2), (4), (6) and (8)) we find a statistically significant and negative effect (semi-elasticities between, −33% and 36%). This strong evidence leads to the adoption of hypothesis C that the revelation of the Panama Papers scandal as a global corruption increasing event leads to fewer FDI flows. This supports the sand the wheels hypothesis, that in general, corruption is seen as a negative and business harming process.

Hypothesis D, regarding the OECD Anti Bribery Convention, is again not so clear. In the specification with the COC, the results show no statistical significance for the dummy variable, but in the models with the corruption distance, we find statistical significance. Due to these non-robust findings, hypothesis D can neither be accepted nor rejected, as it seems this effect is highly dependent on model specification. On the other hand, one could argue, that this shows weak evidence again in favor of the sand the wheels hypothesis.

Regarding the effects of our control variables, most were as expected. GDP and the population of the target country are statistically significant and positively correlated in FDI inflows, confirming the theory, that market size is an attractor of FDI. In models without the OECD anti-bribery dummy, agglomeration effects are also statistically significant and positively correlated, showing some evidence that, target countries with higher FDI stocks attract more FDI.

The trade openness of the target country is statistically significant in all models and shows the expected positive sign. This is again in line with theoretical expectations that, countries with higher trade openness attract more FDI. This is especially the case with regards to vertical FDI, which is the main type of FDI within the OECD.

The dummy for the Eurozone is also statistically significant and positively correlated with FDI flows in all models, indicating that when both countries are members of the Eurozone, they engage in more bilateral FDI with each other. Finally, our results show no significance when it comes to the variables for patents and corporate tax. Therefore, no clear statement can be made for these variables.

### Robustness checks

Robustness checks are done using GDP per capita instead of population. Although this might introduce some problems concerning multicollinearity the results stay robust. Estimated were models (4) and (8). Our variables of interest stay roughly the same and are also significant. We also estimated model (4) using GDP per capita and OLS but the results are questionable (with an r-squared of circa 34% even with dyadic fixed effects). We do, however, find significance for our corruption variables (except COC of the host country) and the signs are also the same. One has to keep in mind, however, that we are estimating in the presence of heteroskedasticity and fixed effects. All these estimations can be found in the Appendix.

## Policy conclusions and further research

This analysis set out to answer the question of whether corruption matters for FDI flows in OECD economies or if subtle differences are not enough, and, if it matters, in which way does it matter. We began by discussing the theoretical foundation underlying corruption research, namely the two differing views of the grease the wheels and the sand the wheels hypotheses. We presented arguments for both sides and presented the relevant empirical literature and its findings. Here, we discovered that in the empirical literature too, the ambiguity of the theoretical arguments can be found. Evidence was presented for both theoretical views. In the next step, the FDI literature was shortly discussed and robust determinants for FDI were identified (based on Faeth (2009), Blonigen and Piger (2014) as well as Eicher et al. (2012)). After stating the hypotheses based on the sand the wheels view, the gravity model was discussed and the theoretical foundation supporting the use of the fravity model (by Larch et al. (2017) in FDI research. A description of the data, the control variables, and the estimation method followed and resulted in a specification of the model. Subsequently, the results were presented. We find evidence that corruption does have complex effects on FDI flows. For target countries of FDI, corruption seems to be positively correlated, whereas for source countries of FDI, we find a negative correlation. Furthermore, one finds strong evidence that the revelation of the Panama Papers scandal resulted in an overall drop in FDI flows. Evidence regarding the OECD Anti-Bribery convention was not as strong and only showed significance in two out of four models, but the expected positive correlation with FDI flows was shown. The concept of corruption distance showed no significance in this dataset.

Arguing that target countries should increase their perceived levels of corruption to attract more FDI goes against common sense. There are more effects of corruption than just increasing FDI inflows. An argument that can be made, however, is that target countries of FDI should focus on other projects, as their corruption levels do not seem to deter FDI. Therefore, focusing on improving infrastructure or generating a business-friendly environment (possibly through reducing corporate taxation, for evidence see Baier and Welfens (2019)) seems to be the way to move forward for these countries. It is also worth noting that FDI is usually associated with positive spill-over effects not just in the technological plane but also in the cultural plane. Therefore, assuming that FDI mainly runs from richer, less corrupt countries to poorer, more corrupt countries, one can argue that by having these FDI inflows some of the company cultures of the MNCs in less corrupt countries might merge with the company culture of the more corrupt countries. Of course, the other side to this argument is simply that in these more corrupt countries, MNCs can more easily engage in corrupt behavior with less fear of getting caught or facing the ramifications of the corruption and therefore there would be no positive cultural spillover effects.

Source countries of FDI on the other hand should indeed look for ways to reduce the corruption prevalent in their countries, as here a reduction in perceived corruption levels correlates with an increase in FDI inflows. A possible explanation for this could be that source countries are generally richer and richer countries tend to have lower corruption levels. Moreover, in these countries the ramifications for corrupt behavior are usually bigger than in corrupt countries, especially regarding media attention, be it traditional media or social media. Another reason might be stronger institutions and a stronger rule of law in these countries.

The results of the revelation of the Panama Papers scandal were very clear. This indicates that, generally, corruption is not seen as something positive for business. An increase in overall perceived corruption, especially in conjunction with an increased focus on the part of the media, society, and politics on the problem of corruption, resulted in a drop in total FDI flows. One argument could be that, the revelation of the Panama Papers scandal showed MNCs that, corrupt activities are not as secret as they might have assumed and the pressure from the public and policymakers resulted in them engaging in less FDI that involved corrupt behavior. The argument for the other side might be that, MNCs were not aware of these widespread levels of corruption in countries with very low perceived corruption levels, resulting in MNCs adjusting their behavior and reevaluating their FDI decisions in the face of these newly uncovered events.

This study is of course not all-embracing. Some topics which can be expanded in future research are for example the country group considered. The OECD is a relatively homogenous group of mostly rich and well-developed countries. Some of the characteristics of these countries are strong institutions and relatively moderate to low corruption levels. It would certainly be of interest to see how FDI flows both into and from lesser developed countries react to corruption. Another consideration is that different measures of corruption could be used. Generally, it would be of interest for future research to use alternative corruption measures such as Transparency International’s Corruption Perception Index.

There could also be special problems in sectors with international industry interdependency, e.g. sectors with a “follow the leader” investment pattern. Such a parallel FDI pattern has not been analyzed here – mainly because of a lack of available sectoral bilateral FDI stock data (and also some problems with the availability of FDI flow data). However, to the extent that the databases of the OECD and the World Bank should improve in relevant fields, future research could look into this issue.

As regards US FDI, a special role in 2018–2019 could come from specific aspects of the Trump Administration’s tax reform in 2017 which has reduced incentives to keep profits made in foreign subsidiaries offshore. It is, for example, unclear whether high profits retained abroad have an impact on effective outward FDI flows of US multinational companies. These aspects could also be covered in future research.

At the bottom line, one clearly can state that the empirical analysis gives crucial new insights into FDI dynamics in the context of an augmented FDI gravity equation. Some of the standard gravity variables were confirmed and new insights into the dynamics of corruption and FDI have been developed. These insights could also be useful for policymakers eager to stimulate FDI inflows as part of a broader supply-side based strategy for overcoming the corona shocks of 2020.

## Data Availability

All data is available online for free.

## Appendix

Table 6 and 7

**Table 6:** PPML dyadic fixed estimation with GDP per capita variables

Variables	(1)	(2)
	Model 1	Model 2
ln_target_gdp	4.892***	4.364**
	(1.848)	(1.846)
ln_origin_gdp	2.506	2.123
	(1.718)	(1.781)
ln_target_gdppc	−3.831*	−3.486*
	(1.968)	(1.957)
ln_origin_gdppc	−2.224	−1.745
	(1.760)	(1.781)
ln_agglo_l1	0.208	0.197
	(0.132)	(0.135)
ln_patents_target	0.216	0.158
	(0.143)	(0.144)
ln_patents_origin	0.0151	0.0814
	(0.120)	(0.123)
target_tax	−0.00367	0.00180
	(0.00990)	(0.0106)
origin_tax	−0.000347	−0.00270
	(0.0104)	(0.0108)
openness	1.981***	1.791***
	(0.372)	(0.364)
ea_dummy	0.235**	0.218*
	(0.119)	(0.126)
coc_l1_target	0.496**	
	(0.204)	
coc_l1_origin	−0.543**	
	(0.256)	
panama_event	−0.416*	−0.390*
	(0.225)	(0.227)
oecd_ab_target	0.304	0.328*
	(0.196)	(0.194)
coc_diff_l1		0.228
		(0.148)
Constant	−55.40***	−50.53***
	(9.591)	(10.21)
Observations	14,626	14,626
R-squared	0.562	0.561

Robust standard errors in parentheses

^***^ *p* < 0.01, ** *p* < 0.05, * *p* < 0.1

**Table 7:** OLS dyadic fixed effect regression, zero flows set to 1$

Variables	(1)
	Model 1
ln_target_gdp	19.35***
	(3.528)
ln_origin_gdp	−2.645
	(3.152)
ln_target_gdppc	−18.48***
	(3.466)
ln_origin_gdppc	6.480**
	(3.231)
ln_agglo_l1	−0.221
	(0.317)
ln_patents_target	0.682**
	(0.276)
ln_patents_origin	0.346
	(0.250)
target_tax	−0.0821***
	(0.0278)
origin_tax	−0.0521*
	(0.0287)
openness	1.400
	(0.884)
ea_dummy	−0.313
	(0.490)
coc_l1_target	1.680***
	(0.626)
coc_l1_origin	−0.581
	(0.595)
panama_event	−8.513***
	(1.255)
oecd_ab_target	1.044**
	(0.453)
Observations	15,408
R-squared	0.349

Robust standard errors in parentheses

^***^ *p* < 0.01, ** *p* < 0.05, * *p* < 0.1

Correlation table part 1 of 3.

**Table:**

	inflow	ln_target_gdp	ln_origin_gdp	ln_target_gdppc	ln_origin_gdppc
inflow	1				
ln_target_gdp	0.174***	1			
ln_origin_gdp	0.155***	−0.00366	1		
ln_target_gdppc	0.142***	0.273***	0.0725***	1	
ln_origin_gdppc	0.141***	0.0255**	0.297***	0.133***	1
ln_pop_target	0.114***	0.901***	−0.0365***	−0.173***	−0.0337***
ln_pop_origin	0.0956***	−0.0155	0.900***	0.0145	−0.149***
ln_agglo_l1	0.226***	0.849***	0.0258**	0.399***	0.0936***
ln_patents_target	0.132***	0.899***	−0.0145	0.252***	−0.00469
ln_patents_origin	0.126***	−0.0112	0.895***	0.0173*	0.235***
target_tax	0.115***	0.558***	−0.0785***	0.127***	−0.188***
origin_tax	0.0941***	−0.0235**	0.513***	−0.138***	0.135***
openness	0.0218**	−0.528***	0.0442***	0.116***	0.0874***
ea_dummy	0.0445***	−0.0985***	−0.0955***	0.174***	0.183***
coc_l1_target	−0.0830***	−0.0581***	−0.0121	−0.724***	0.0248**
coc_l1_origin	−0.0990***	−0.00697	−0.0561***	0.0295***	−0.700***
panama_event	0.0163*	0.0137	0.0223**	0.0991***	0.0900***
oecd_ab_target	0.0371***	0.0517***	0.0491***	0.238***	0.196***
coc_diff_l1	−0.120***	−0.0626***	−0.0615***	−0.233***	−0.129***

Correlation table part 2 of 3.

**Table:**

	ln_pop_target	ln_pop_origin	ln_agglo_l1	ln_patents_target	ln_patents_origin
inflow					
ln_target_gdp					
ln_origin_gdp					
ln_target_gdppc					
ln_origin_gdppc					
ln_pop_target	1				
ln_pop_origin	−0.0224**	1			
ln_agglo_l1	0.689***	−0.0160*	1		
ln_patents_target	0.807***	−0.0129	0.663***	1	
ln_patents_origin	−0.0192*	0.820***	−0.0129	−0.0115	1
target_tax	0.514***	0.00475	0.314***	0.524***	−0.00365
origin_tax	0.0383***	0.469***	−0.124***	0.0110	0.522***
openness	−0.594***	0.00588	−0.169***	−0.538***	0.00151
ea_dummy	−0.180***	−0.183***	−0.0302***	−0.147***	−0.147***
coc_l1_target	0.268***	−0.0239**	−0.181***	−0.137***	−0.0193*
coc_l1_origin	−0.0205*	0.261***	0.0287***	−0.0156	−0.118***
panama_event	−0.0302***	−0.0175*	0.0939***	−0.0197*	−0.0263**
oecd_ab_target	−0.0547***	−0.0386***	0.241***	0.0216**	−0.0327***
coc_diff_l1	0.0409***	−0.00533	−0.106***	−0.0948***	−0.0890***

Correlation table part 3 of 3.

**Table:**

	target_tax	origin_tax	openness	ea_dummy	coc_l1_target	coc_l1_origin	panama_event	oecd_ab_target	coc_diff_l1
inflow									
ln_target_gdp									
ln_origin_gdp									
ln_target_gdppc									
ln_origin_gdppc									
ln_pop_target									
ln_pop_origin									
ln_agglo_l1									
ln_patents_target									
ln_patents_origin									
target_tax	1								
origin_tax	0.225***	1							
openness	−0.454***	−0.121***	1						
ea_dummy	−0.0628***	−0.0627***	0.137***	1					
coc_l1_target	−0.170***	−0.0544***	−0.0128	−0.0660***	1				
coc_l1_origin	−0.0508***	−0.148***	0.0403***	−0.0657***	−0.000370	1			
panama_event	−0.191***	−0.188***	0.0777***	0.0390***	0.00602	0.0405***	1		
oecd_ab_target	−0.359***	−0.312***	0.187***	0.0993***	0.0188*	0.0517***	0.117***	1	
coc_diff_l1	−0.0724***	−0.0608***	−0.0220**	−0.0565***	0.282***	0.155***	−0.0127	0.0122	1

## Footnotes

1 $\mathrm{F}= G\frac{{m}_{1}*{m}_{2}}{{r}^{2}}$, where m = mass, r = distance, and G = gravitational constant.

2 For target country > −0.7 for the host country ~ −0.7.

3 Semi elasticities between 6.1% and 6.7%

4 Semi elasticities between 26 and 31%

5 Changes in significance levels compared to models (1) to (4) in brackets.

## Code availability

Statistic software used is Stata, PPML command by: J.M.C. Santos Silva & Silvana Tenreyro, 2015. "PPML: Stata module to perform Poisson pseudo-maximum likelihood estimation," Statistical Software Components S458102, Boston College Department of Economics.
